# Proteomics and Interspecies Interaction Analysis Revealed Abscisic Acid Signalling to Be the Primary Driver for Oil Palm’s Response against Red Palm Weevil Infestation

**DOI:** 10.3390/plants10122574

**Published:** 2021-11-25

**Authors:** Nazmi Harith-Fadzilah, Su Datt Lam, Mohammad Haris-Hussain, Idris Abd Ghani, Zamri Zainal, Johari Jalinas, Maizom Hassan

**Affiliations:** 1 Institute of Systems Biology, Universiti Kebangsaan Malaysia, Bangi 43600, Selangor, Malaysia; P92950@siswa.ukm.edu.my (N.H.-F.); zz@ukm.edu.my (Z.Z.); 2 Department of Applied Physics, Faculty of Science and Technology, Universiti Kebangsaan Malaysia, Bangi 43600, Selangor, Malaysia; sudatt@ukm.edu.my; 3 Institute of Structural and Molecular Biology, University College London, London WC1E 6BT, UK; 4 Department of Biological Sciences and Biotechnology, Faculty of Science and Technology, Universiti Kebangsaan Malaysia, Bangi 43600, Selangor, Malaysia; p100206@siswa.ukm.edu.my (M.H.-H.); idrisyatie@ukm.edu.my (I.A.G.); Johari_j@ukm.edu.my (J.J.)

**Keywords:** *Elaeis guineensis*, *Rhynchophorus ferrugineus*, proteomics, plant-insect interactions, herbivory, shotgun proteomics

## Abstract

The red palm weevil (RPW; *Rhynchophorus ferrugineus* Olivier (Coleoptera Curculionidae)) is an invasive insect pest that is difficult to manage due to its nature of infesting the host palm trees from within. A holistic, molecular-based approach to identify proteins that correlate with RPW infestation could give useful insights into the vital processes that are prevalent to the host’s infestation response and identify the potential biomarkers for an early detection technique. Here, a shotgun proteomic analysis was performed on oil palm (*Elaeis guineensis*; OP) under untreated (control), wounding by drilling (wounded), and artificial larval infestation (infested) conditions at three different time points to characterise the RPW infestation response at three different stages. KEGG pathway enrichment analysis revealed many overlapping pathways between the control, wounded, and infested groups. Further analysis via literature searches narrowed down biologically relevant proteins into categories, which were photosynthesis, growth, and stress response. Overall, the patterns of protein expression suggested abscisic acid (ABA) hormone signalling to be the primary driver of insect herbivory response. Interspecies molecular docking analysis between RPW ligands and OP receptor proteins provided putative interactions that result in ABA signalling activation. Seven proteins were selected as candidate biomarkers for early infestation detection based on their relevance and association with ABA signalling. The MS data are available via ProteomeXchange with identifier PXD028986. This study provided a deeper insight into the mechanism of stress response in OP in order to develop a novel detection method or improve crop management.

## 1. Introduction

The red palm weevil (RPW, *Rhynchophorus ferrugineus* Olivier (Coleptera Curculionidae) is an invasive insect pest for various palm species. RPW is endemic in South and Southeast Asian countries, and has become a prevalent pest problem in Asian countries, the Middle East, and the Mediterranean [1]. RPW infestation can inflict mortality on host trees. Examples of economically significant palms affected by RPW are Canary Island date palm (*Phoenix canariensis*), date palm (*Phoenix dactylifera*), oil palm (*Elaeis guineensis,* OP), coconut (*Cocos nucifera*), and sago (*Metroxylon sago*) [1]. RPW attacks the trunk and the crown of the host palm. In coconut palms, RPW also infests from the roots [2]. They usually exploit wounded or pruned parts as these parts produce volatile organic compounds (VOCs) that can be detected by RPW [3].

The progression of RPW infestation is a rapid process but dependent on the size of the infested palm and the infested site as well. In a mature Canary Island date palm, the infestation often occurs in the crown where the infestation is asymptomatic for three months. Once the physical symptoms appear, significant decrowning is achieved in a matter of a week [4]. In date palm, the infestation more commonly occurs in the trunk. The infestation could occur for several RPW lifecycles and the extent of the damage could only be evaluated by cutting open suspected bore holes [5]. In our previous study on two-year-old infested oil palms, the artificial infestation (on the crown) took four weeks to exhibit a sign of infestation and only nine weeks to achieve significant decrowning [6]. Therefore, it is important to detect infested trees before physical symptoms appear as significant damage could occur before the infestation can be detected visually. In Malaysia, the RPW infestation problem is prevalent in coconut trees. However, adult RPWs have already been detected in oil palm plantations, posing potential threats to the most important economic crop of the country [7].

Plants have a systemic hormone signalling mechanism called systemic acquired resistance (SAR) in which the plant tissues attacked by insects or pathogens produce plant hormones that travel throughout the plant and induce a defence response [8,9]. This mechanism allows the plant to be more resistant to further attacks. The established SAR hormones are jasmonic acid (JA) and salicylic acid (SA) as they are known to induce gene expressions related to plant defence and the release of defensive VOCs [10,11]. However, other hormones, such as abscisic acid (ABA) and ethylene (ET), are also found to be associated with plant’s defence against biotic stresses despite their primary roles in abiotic stress acclimatisation and growth, respectively [12,13,14]. The effects of SAR hormone signalling would result in a different protein expression profile of RPW-infested trees compared to non-infested trees. Those differentially expressed proteins could potentially be harnessed as molecular biomarker candidates that can be developed into molecular-based RPW-infested trees detection methods.

The plant’s mechanism of recognising threats and consequently inducing the synthesis of SAR-related hormones is mostly unknown. Nonetheless, it has been established that plants possess pattern recognition receptors (PRRs) that recognise elicitor molecules and subsequently induce the defence response [15]. These elicitor molecules can be secreted from microbial pathogens (microbial-associated molecular patterns, MAMPs) [16], insect herbivores (herbivory-associated molecular patterns, HAMPs) [17], or endogenous molecules that are released to extracellular space due to tissue damage (damage-associated molecular patterns, DAMPs) [18]. For RPW, several compounds that might be vital for herbivory are identified and they can mediate the host’s defence response [19,20]. The contemporary knowledge of PRRs is still insufficient to correlate a particular PRRs to a particular set of elicitor molecules. Broadly speaking, PRRs that are currently known to mediate responses to MAMPs, HAMPs, or DAMPs possess leucine-rich repeat (LRR) domain [21]. Identifying PRRs that play a pivotal role in mediating the interaction with a pathogen or an insect herbivores could provide in-depth knowledge regarding the plant’s defence mechanism and clues to improve the plant’s pest and pathogen tolerance via genetic engineering [22]. An in-silico approach via molecular docking could potentially direct the research towards finding these PRRs by predicting potentially interacting molecules and receptor proteins between the secretions of insect pests or pathogens and the host plant.

In this study, proteomic analysis was utilised to identify differentially expressed protein in RPW-infested OP trees at three infestation stages: no physical symptoms and no physiological changes, no physical symptoms and presence of physiological changes, and presence of both physiological changes and physical symptoms. A molecular docking approach was utilised to predict the interaction between RPW larva ligands and OP receptors that mediated the SAR response. A subset of proteins relevant to response against RPW herbivory were proposed as candidate biomarkers for a molecular-based method of detecting RPW-infested OP trees.

## 2. Results

The proteomic expression profiles were compared among the control, wounded, and infested groups. On each chosen week, differentially expressed proteins were identified by comparing control and infested group (C/I), control and wounded group (C/W), and wounded and infested group (W/I).

### 2.1. Patterns of Protein Expressions

**Figure 1:**
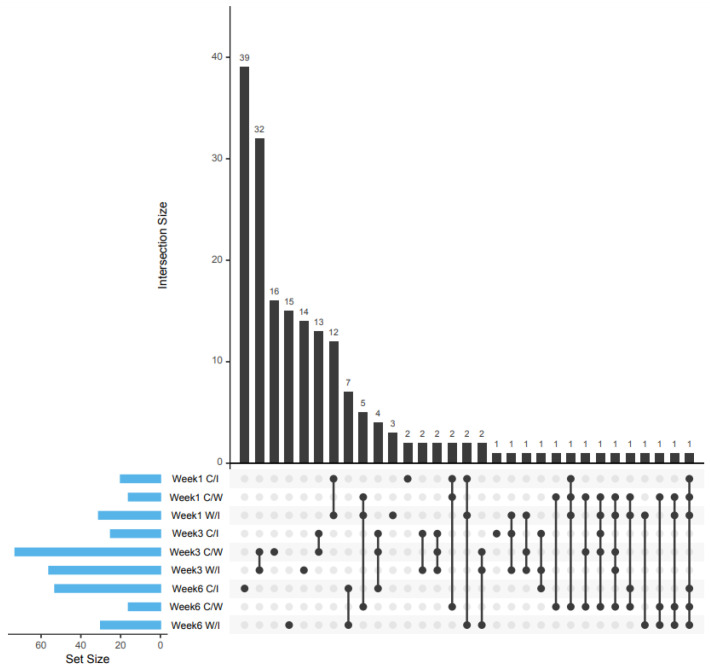
UpSet diagram for comparing the differentially-expressed proteins reported among the control and infested comparison (C/I), control and wounded comparison (C/W) and wounded and infested comparison (W/I) groups on the first, third and sixth week post RPW infestation.

There were 39, 92, and 97 differentially expressed proteins in the first, third, and sixth weeks post-RPW infestation, respectively (Tables S1–S3). It was noteworthy that the underexpressed proteins in the C/I comparison groups were overexpressed in the W/I comparison groups and vice versa. On the first week, most of the differentially expressed proteins were found between the C/I and W/I comparison group with 12 proteins. In contrast, a majority of the differentially expressed proteins on the third week were shared between the C/W and W/I comparison groups with 32 proteins. On the sixth week post-RPW infestation, a majority of the differentially expressed proteins were found in C/I comparison group followed by W/I with 39 and 14 proteins respectively. The patterns of differentially expressed proteins patterns lacked consistency when compared across the first, third, and sixth weeks post-RPW infestation (Figure 1). There is no protein that was consistently differentially expressed from the first to the sixth week in any of the C/I, C/W, or W/I comparison groups. Only a handful of proteins had a consistently differential expression between the first and the third weeks or between the third and the sixth weeks.

The KOBAS enrichment analysis of the three comparison groups on the three time points found significant overlapping of KEGG pathways among them (Table 1). Among the frequently appearing enrichments were photosynthesis, carbon metabolism, metabolic pathways, and secondary metabolite biosynthesis. In addition, there was no specific pathways found related to stress response in W/I or C/I groups across all three time points, suggesting high similarities of processes that were differentially expressed in control, wounded, and infested groups. Pathways that could be relevant to the plants’ response to insect attacks were photosynthesis and secondary metabolite biosynthesis, because photosynthesis was previously reported to drop in the third week post-infestation [6] and secondary metabolites that were differentially expressed might play a role in defence.

### 2.2. Literature Research on Differentially Expressed Proteins

Due to the limited information derived from KOBAS enrichment, we performed literature research to select candidate biomarkers. Only differentially expressed proteins in W/I but not in C/I and C/W were selected to ensure that the protein expression patterns were attributed to RPW infestations. These proteins were then filtered based on their relevance to the plant’s response to insect attacks and their categories (photosynthesis, growth, and stress response). Furthermore, the response to SAR-related hormone signals based on previous research was also highlighted for each protein of interest. A total of 24 proteins were found to be associated with the plant’s response to insect attacks (Table 2).

Seven photosynthesis-related proteins were differentially expressed proteins across the three time points. The majority of these proteins were found in the first week post-infestation. All seven proteins were upregulated (Log_2_ W/I < −1.5) in the infested group. Only four growth process proteins were identified. Two of them were significantly overexpressed: 3-mercaptopyruvate sulfurtransferase (3-MST) and thioredoxin M-type (TRXM). The other two proteins which were significantly underexpressed (Log_2_ W/I > 1.5) were PsbP domain-containing protein 6 (PPD6) and NADPH-dependent thioredoxin reductase (NTRB).

On the other hand, 16 differentially expressed proteins were grouped under ‘stress response’. This category made up the majority of the proteins of interest. Ten proteins showed overexpression whereas six showed underexpression under RPW infestation across all three time points. The sixth week had the largest number of proteins differentially expressed under this category with eight proteins, whereas the third week had the lowest number with only three.

### 2.3. Modelling and Docking Analysis

A total of 34 LRR receptors were successfully modelled based on eight templates and a docking analysis was performed against four RPW ligand compounds reported previously to induce a plant’s immune response: 5-methoxytriptamine (5-MT), γ-aminobutrytic acid (GABA), aminooxyacetic acid (AAO), and putrescine (PUT) (Table 3). The docking position of RPW ligands on the OP proteins was compared to the docking position of the corresponding template protein’s ligand on the template protein. This was done to overcome the knowledge gap present in the RPW molecules and OP proteins of which the interacting molecules between these two species were unknown. Based on the fact that the interactions between the template and its ligand had been proven, we postulated that the OP protein with homology to that template protein would possess the same ligand interaction site. Thus, we predicted that RPW ligands with similar docking positions as to the template ligand were putatively interacting. Out of the eight templates, five of them (PDB ID 5UV4, 6BRJ, 3UIM, 4Z63, and 6BSD) had modelled proteins having RPW ligands binding position overlapping or in very close proximity to their corresponding template ligand.

The binding affinity between the OP protein and RPW ligand was also compared between the template protein and template ligand. Most of the interactions between the OP receptors and RPW ligands had lower binding affinity scores compared to the binding affinity between the template and the template’s ligand (Table 4). Out of the five modelled templates with predicted interactions, two had RPW ligand binding positions overlapping the template ligand (PDB ID 5UV4 and 6BRJ). In contrast, the other three (PDB ID 3UIM, 4Z63, and 6BSD) had a ligand binding position close to their corresponding template ligand.

#### 2.3.1. Overlapping Binding Region

Protein PDB 5UV4 is a sucrose-induced receptor kinase 1 and chosen as the template for 11 OP proteins. XP_010942956.1 (LRR receptor-like serine/threonine-protein kinase FEI 1 (LRR-FEI1)) was chosen as representative due to its strongest binding energy. LRR-FEI1 interacted with AAO and 5-MT (Figure S1). The interactions between 5-MT and LRR-FEI1 consisted mostly of Van der Waals forces followed by conventional hydrogen bonds, and the presence of Pi-sigma and alkyl bonds (Figure S2). The interactions between LRR-FEI1 and AAO were primarily conventional hydrogen bonds and Van der Waals forces.

Three proteins were modelled after epithelial discoidin domain-containing receptor 1 (DDR1, PDB ID 6BRJ). They were all identified as probable inactive receptor kinase At5g58300 (At5g58300). XP_010931393.1 was arbitrarily selected as a representative. This protein interacted with ligand 5-MT (Figure S3). Hence, 5-MT interacted with 13 residues of At5g58300 with Van der Waals, conventional hydrogen bonds, and alkyl interactions.

#### 2.3.2. Close Proximity Binding Region

**Figure 2:**
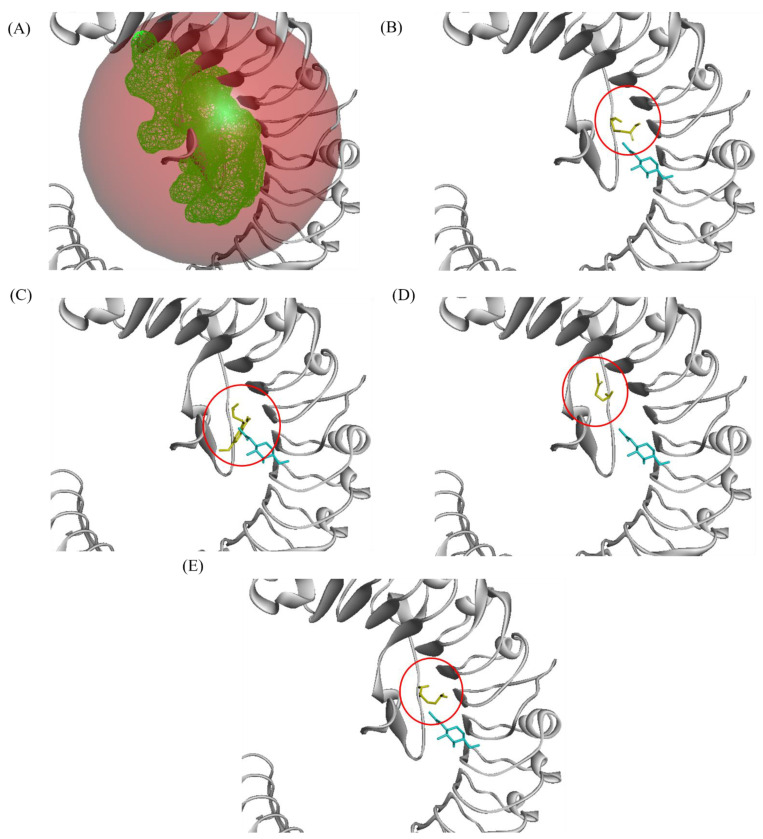
Predicted ligand position docking position on phytosulfokine receptor 2 (PSKR2). (**A**) Predicted docking site highlighted in green mesh surface. (**B**) GABA ligand binding position (yellow) compared to template ligand (blue). (**C**) 5-MT ligand binding position (yellow). (**D**) AAO ligand binding position. (**E**) PUT ligand binding position. 5-MT: 5-methoxytriptamine; AAO: aminooxyacetic acid; PUT: putrescine.

**Figure 3:**
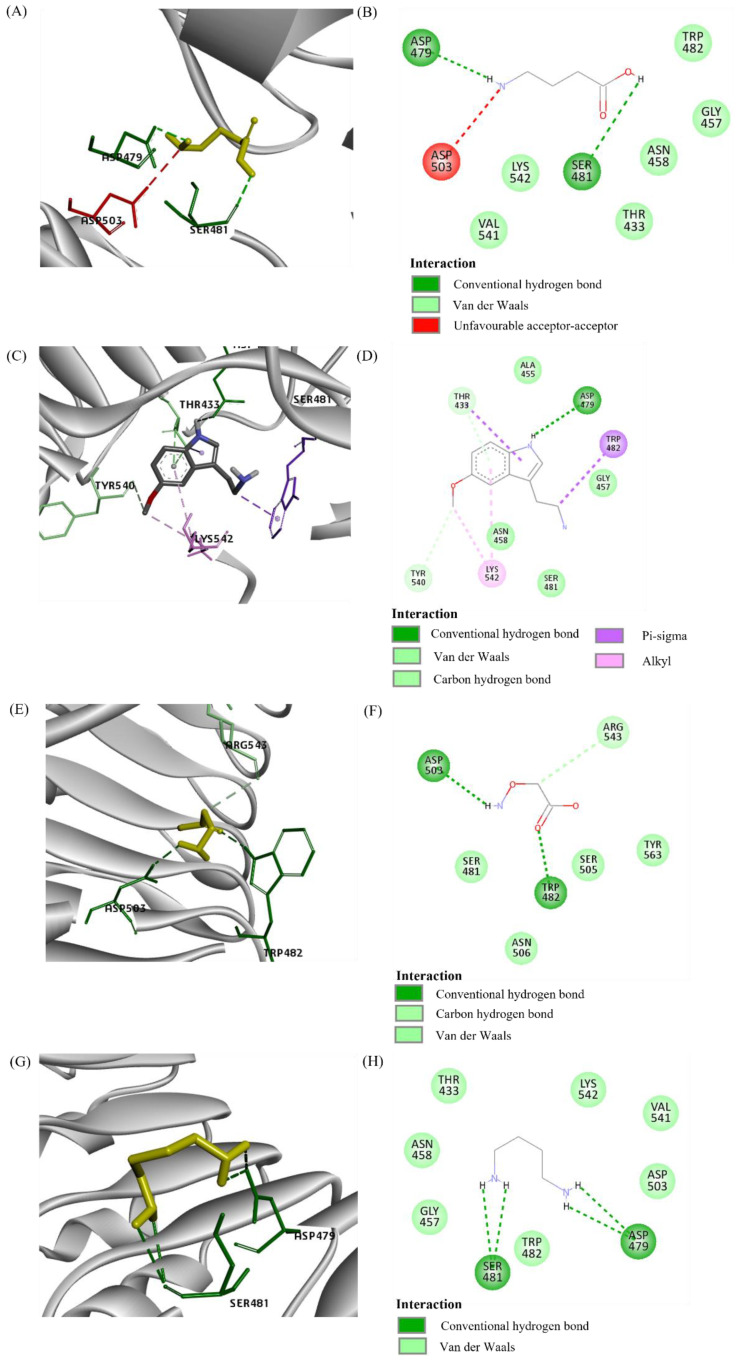
Molecular interaction among RPW ligands and PSKR2. (**A**) 3D interaction diagram between GABA (yellow) and PSKR2. (**B**) 2D interaction diagram between GABA and PSKR2. (**C**) 3D interaction diagram between 5-MT and PSKR2. (**D**) 2D interaction diagram between 5-MT and PSKR2. (**E**) 3D interaction diagram between AAO and PSKR2. (**F**) 2D interaction diagram between AAO and PSKR2. (**G**) 3D interaction diagram between PUT and PSKR2. (**H**) 2D interaction diagram between PUT and PSKR2. 5-MT: 5-methoxytriptamine; AAO: aminooxyacetic acid; PUT: putrescine.

Three LRR receptors were proteins modelled using PDB ID 4Z63. XP_010929346.1 (namely phytosulfokine receptor 2 (PSKR2)) interacted with all ligands tested (Figure 2). The majority of the interactions were Van der Waals, followed by hydrogen bonds (Figure 3). However, there were alkyl and pi-sigma interactions between 5-MT and PSKR2, and an unfavourable acceptor–acceptor interaction between GABA and PSKR2.

**Figure 4:**
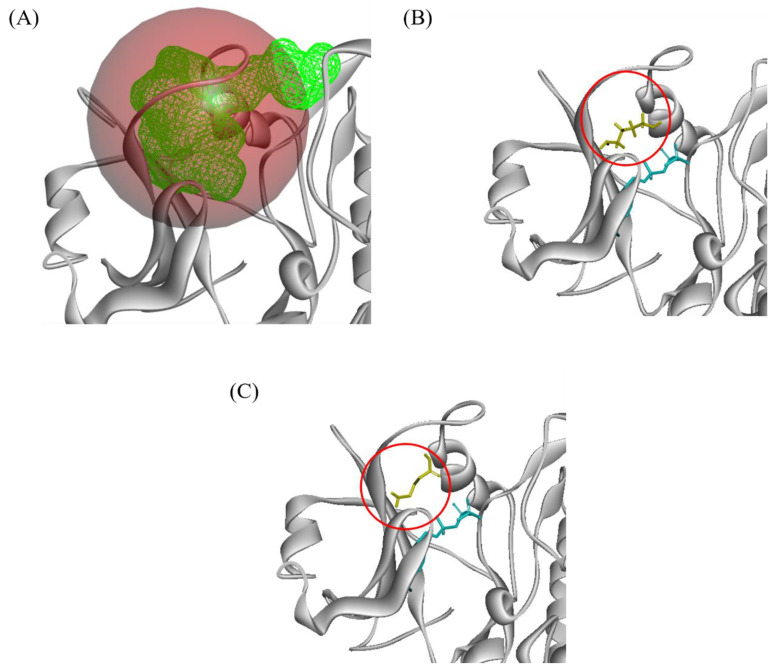
Predicted Red Palm Weevil (RPW) ligand binding position on oil palm’s (OP) Nuclear protein interacting kinase (NIK) protein. (**A**) predicted ligand docking site highlighted in green mesh surface. (**B**) GABA ligand binding position (yellow) in comparison to template ligand (blue) (**C)** PUT ligand binding position (yellow) in comparison to template ligand (blue).GABA: γ-aminobutyric acid; PUT: Putrescine.

**Figure 5:**
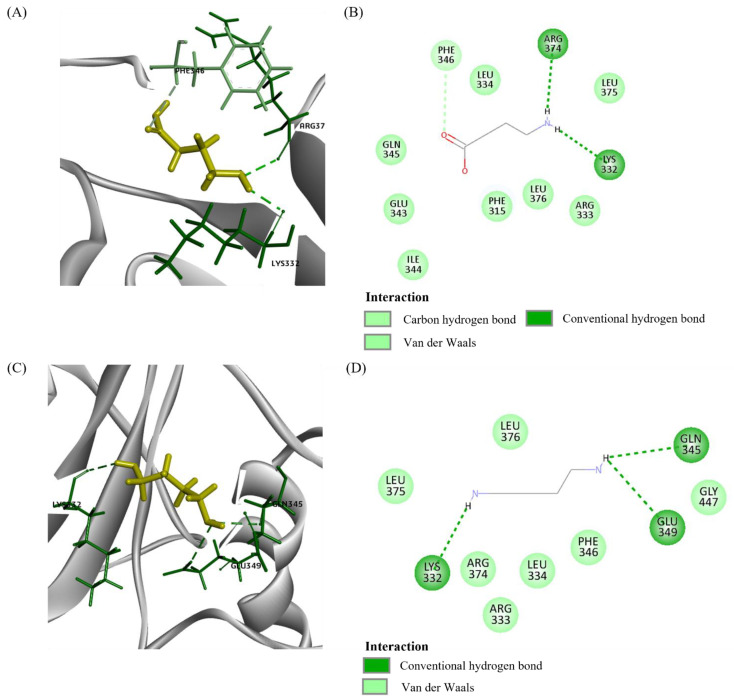
Molecular interaction among RPW ligands and NIK. (**A**) 3D interaction diagram between GABA (yellow) and NIK. (**B**) 2D interaction diagram between GABA and NIK. (**C**) 3D interaction diagram between PUT and NIK. (**D**) 2D interaction diagram between PUT and NIK. GABA: γ-aminobutyric acid; PUT: Putrescine.

Seven LRR receptors were modelled using brassinosteroid insensitive 1-associated receptor kinase (BAK1) (PDB ID 3UIM). XP_010942232.1 (nuclear shuttle protein (NSP) interacting kinase (NIK)) interacted with two ligands: GABA and putrescine (Figure 4 and Figure 5). GABA interacted with 11 protein residues whereas putrescine interacted with 10 protein residues. For both ligands, their interactions were primarily Van der Waals and hydrogen bonds.

PDB 6BSD (DDR1) was used to model XP_010916177.1 (probable inactive receptor kinase At2g26730 (At2g26730)). At2g26730 interacted with 5-MT (Figure S4). We found 16 At2g26730 residues interacting with 5-MT, and the majority of the interactions comprised of Van der Waals, followed by alkyl interactions. There was only one conventional hydrogen interaction and one Pi-sigma interaction occurring in this interaction.

## 3. Discussion

Proteomic analysis was performed on OP leaves under control, wounding by drilling, and artificial infestation with RPW larvae conditions. The analysis was also performed across three different weeks post-infestation based on the physical symptoms and the changes in the photosynthetic activity observed in the RPW-infested trees in our previous study [6]. The first week of the larvae infestation was the stage where there was no symptom of infestation. On the third week, the RPW-infested trees showed decline in photosynthetic activity, but no physical symptoms of infestation were observed. On the sixth week, the infested trees showed signs of palm decrowning and a significant decline in photosynthetic activity.

Initial observation of the differentially expressed proteins showed little consistency between expression patterns of the control, wounded, and infested group across the three time points. Yet, further pathway enrichment analysis showed a large similarity of enriched process across the C/I, C/W, and W/I comparison groups. The result suggests a significant similarity between response to mechanical wounding by drilling and response to stem-boring pest (like RPW).

### 3.1. Photosynthesis-Related Proteins

Upwards trends of protein expression related to photosynthesis might suggest an increase in photosynthesis activity. This highlighted a discrepancy with our previous study which observed a decline in photosynthetic activity with prolonged RPW infestation [6]. Another research that studied the influence of protein expression patterns of RPW on date palm (*Phoenix dactylifera)* reported the underexpression of proteins related to photosynthesis on the third day post-infestation [65]. The highest proportion of proteins differentially expressed under RPW infestation in that study was ribulose biphosphate carboxylase (Rubisco). In contrast, Rubiscos were not differentially expressed in the infested or the wounded group in this study across all three time points. Biological factors of different trees and climates could factor in the observed discrepancy.

On the first week post-infestation, four photosynthesis-related proteins were overexpressed, namely translation initiation factor IF3-2 (IF3-2), photosystem I P700 chlorophyll a apoprotein A1 (PsaA), photosystem II CP47 reaction centre protein (PsbB), and the cytochrome b6. It must be noted that at this time point, the photosynthesis activity of infested OP trees was not significantly different from the wounded and control groups [6]. Hence, the overexpression of these four proteins may be vital for maintaining photosynthetic activity while the RPW larva herbivory was damaging the tree. IF3-2 played a significant role in chloroplast development. The decline in IF3-2 activity deformed the morphology of chloroplast and leaves [23]. Therefore, the overexpression of IF3-2 on the first week post-infestation might be associated with the damage caused by the larva RPW herbivory activity. The PsaA and PsbB were involved with photosynthesis processes in photosystem I (PSI) and II (PSII), respectively. PsaA serves a primary role as an electron donor in photosystem I [66]. A more recent study found that loss-of-function mutation of PsaA caused deformities in chlorophyl due to impaired photosystem I and II mechanism [24]. In addition, the expression of PsaA could be induced via ABA signalling [25]. On the other hand, PsbB binds chlorophyll structures together, thus mediating the photosynthesis light reaction [26]. Previous studies reported that the expression of PsbB could be inhibited by JA but induced by ABA signalling [25,26,27]. The cytochrome b6, also known as photosynthetic electron transport B (PetB), mediates electron transfer between PSII and PSI. PetB serves as a component regulating the photosynthetic electron transport and light-harvesting process during photosynthesis [28]. The overexpression of PetB might have an important but undiscovered role in maintaining normal photosynthetic activity under RPW infestation.

In the third week, a significant decline of photosynthetic activity in infested OP trees was observed [6]. At this time point, chlorophyll ab binding protein 5 (CAB5) and 15-cis-phytoene desaturase (PDS) were overexpressed. CAB5 serves as the light receptor and transfers excitation energy into the photosystem [67]. The expression of CAB5 genes and proteins is positively regulated by ABA signalling [47]. However, under ABA accumulation, CAB5 mediates stomatal closure [46]. Thus, CAB5-mediated stomatal closure may have caused the decline in photosynthetic activity of the infested OP trees. PDS is a plasma-membrane localised protein that plays a role in carotenoid biosynthesis [49]. Carotenoid functions to transfer excitation energy to the PSII reaction centre and also helps to protect the integrity of chlorophyll structure via reactive oxygen species (ROS) sequestration, making it vital for protection against oxidative stress [50]. This protein expression could be induced positively by SA signalling [48]. Moreover, it is possible that this gene is also induced by ABA signalling as a previous study reported that ABA treatment on plants was linked to increased carotenoid concentration in plants [48].

Overall, the overexpression of photosynthesis-related proteins could be linked to ABA signalling except for IF3-2 and PetB. Based on the infested OP trees’ protein expressions, OP may produce ABA hormones as a response to RPW infestation. Apart from PsbB, no protein under this category was found to be associated with other phytohormones, such as SA, JA, or ethylene. As ABA signalling is associated with stomatal closure [68], these overexpressed proteins may play a role in the process. However, from photosynthesis-related proteins, only CAB5 is known to mediate stomatal closure.

### 3.2. Growth Related Proteins

In our previous research, the growth of RPW-infested OPs appeared not to be different between wounded and control groups. However, while we inferred that the OP herbivory might affect the host plant’s growth processes, the process was very rapid in that it was not reflected physically in terms of change in height and circumference. Four differentially expressed proteins were related to growth processes. They were found in the third week and sixth weeks only. In the third week, TRXM and 3-MST were overexpressed while NTRB was underexpressed. TRXM has a primary role in maintaining redox homeostasis in the chloroplast, which is vital for meristem maintenance [41,69]. The regulation of ROS levels via redox homeostasis consequently regulates the balance between plant defence and photosynthetic activity [42,43]. In contrast, 3-MST activity was vital in ensuring proper embryo and seed development based on a previous loss-of-function mutant study on *A. thaliana* [40]. However, the 3-MST deficient mutants were not affected in terms of plant’s height growth compared to normal wildtype. The overexpression of 3-MST suggests an unknown role of this protein in the event of stem-boring insect infestation. NTRB has a vital role in ensuring normal shoots and roots growth at the meristems [70]. It is involved in mediating both auxin and nitric oxide signalling in a positive manner, with both hormones stimulating plant growth and development [44,45]. Underexpression of NTRB suggests the growth processes might be reduced.

On the sixth week, PPD6 was underexpressed in the infested group. Gene co-expression analysis found that PPD6 was co-expressed with stress-related genes including high light intensity [71,72]. Furthermore, a study that employed RNA interference assays to disrupt PPD genes expression reported that those plants had retarded growth and increased sensitivity to high light intensity [53]. In addition, the activity of its homologue, PPD5, regulates the ABA signalling response negatively to reduce ROS accumulation in the cell [54].

The overall growth-related protein trend suggests a shift in biological processes that favour survival rather than growth within OP. The mixed response of underexpression of PPD6 but overexpression of TRXM suggested that the ROS levels were being carefully modulated under RPW infestation. This response may be imperative to ensure that the plant’s stress response mechanisms are activated without completely abandoning growth-related processes.

### 3.3. Stress Response Related Proteins

A previous study investigating RPW infestation on coconut reported an increase in enzymatic antioxidants activity and in antioxidant production in the infested trees, suggesting that oxidative stress was inflicted upon them [73]. On the first week, Phospholipid hydroperoxide glutathione peroxidase (PHGPX), remorin, Subtilisin-like protease SBT1.2 (SBT1.2), 2-alkenal reductase (NADP (+)-dependent) (DBR), and dirigent protein 19 (DIR19) were overexpressed in the infested group. PHPGPX protein mitigates the oxidative stress caused by lipid peroxidation [34,74]. In addition, a previous study reported an overexpression of PHGPX gene under JA, SA, ABA, and auxin signalling [75]. That study inferred that those hormones’ signalling mechanisms induced ROS accumulation, and thus PHGPX quenched ROS as a means for oxidative stress protection. Thus, the expression of PHGPX is likely a response to plant hormone signalling. Remorin is a membrane-bound protein component of lipid rafts that mediate plant–microbe interactions and activate the plant’s defence system by recruiting LRR-RLK [76]. It was reported that remorin genes were overexpressed under ABA accumulation conditions in rice plants (*Oryza sativa*) [35], and SA also induced its activity to mediate plasmodesmal closure, preventing virus entry [77]. SBT1.2 degrades misfolded proteins and aids in post-translational modifications of proteins [36]. There is also an additional role of SBT1.2 in regulating stomata development by inhibiting this process, consequently reducing water loss via transpiration [37]. This protein was not known to be modulated by or responded to plant hormones. Nevertheless, the overexpression of SBT1.2 was consistent with the decline in photosynthesis activity due to RPW infestation as observed in our previous study [6]. DBR reduces reactive carbonyls, such as 2-aklenal and oxenes, which are cytotoxic [38]. Its localised activity in chloroplast serves to mitigate photooxidative stress by scavenging those reactive carbonyls present in the organelle [39]. The relationship of this protein with defence against insect attacks and plant hormone response is currently not known. The DBR overexpression suggests a potential role of DBR in mitigating stress factors outside of photooxidative stress. The DIR protein families are involved in both lignin and lignan biosynthesis which is implicated in cell wall remodelling [29]. Lignin has a role in lignification of cell wall which can aid in resisting further pathogen and insect attacks [29]. On the other hand, lignan serves as a source of monolignol storage utilised by lignification in plants [32,33]. DIR genes were overexpressed in pine trees (*Pinus pinaster*) under drought stress [30].

On the third week, PDS was overexpressed but extracellular ribonuclease LE (RNase LE) was underexpressed. The role of PDS was discussed previously in photosynthesis and stress response. RNase-LE has a primary function in hydrolysing phosphodiester bonds on RNA molecules [52]. A previous study reported several extracellular ribonucleases expressed on the systemic part of *A. thaliana* following artificial wounding [51]. Furthermore, some extracellular ribonucleases are members of pathogenesis-related 10 family proteins which aid in the plant’s resistance against virus infections [52]. However, the specific role of RNase LE with regard to plant defence is not currently known, and this protein is also not known to be modulated by any plant’s hormones.

On the sixth week, DIR2, 16.9 kDA, 18.1 kDA, and 22 kDA heat shock proteins (HSP16.9, 18, and 22) were upregulated whereas DIR7, glutathione S-transferase F11 (GSTF11) and 2,3-bisphosphoglycerate-independent phosphoglycerate mutase (PGM-I) were underexpressed. HSPs are molecular chaperones that ensure proper protein folding and protect proteins from misfolding under stressful events [78]. These three enzymes also protect cells from oxidative stress by mitigating H_2_O_2_ accumulation. HSP16.9 activity was found to increase peroxidase, catalase, and superoxide dismutase (SOD) activities in the tobacco plant (*Nicotiana tabacum*). On the other hand, HSP18 was found to be overexpressed under high temperature stress in green pea plants (*Pisum sativum*) [58]. In similar trends, HSP22.7 gene was overexpressed under drought stress in corn (*Zea mays*) [59]. In contrast, a proteomic analysis on RPW-infested date palm trees (*Phoenix dactylifera*) found the overexpression of two 70 kDA HSPs which were not found to be differentially expressed in this study [65]. Due to the sampling time difference between that study and this one, it might be possible that different HSPs are expressed differentially depending on infestation stage. Furthermore, HSPs activities are known to be affected by ABA signalling. ABA accumulation in cells induces heat shock factor activity, which acts as the transcription factors for HSPs, hence promoting HSP biosynthesis [60]. In addition, this HSPs production could also be induced by MeJA as demonstrated in various plants such as tomatoes (*Solanum lycopersicum*), pomegranates (*Punica granatum*), loquats (*Eribotrya japonica*), mangoes (*Mangifera indica*), and guavas (*Psidium guajava*) [79]. Similarly to DIR19, DIR2 and DIR7 have a role in lignin and lignan biosynthesis and may play a vital role in abiotic stress management. The expression of these DIR proteins is influenced by ABA, SA, and MeJA signalling but the specific effects that these hormones have are dependent upon the respective DIR proteins [30,31]. Thus, the mixed responses of two DIR proteins found in this study might be caused by each DIR protein responding differently to hormone signals. GSTF11 conjugates glutathione tripeptides on toxic compounds, detoxifying them [56]. The specific role of GSTF11 is not well known but other GSTFs have been observed to aid in resisting pathogen infection and oxidative stress. For example, GSTF2, 5, 6, and 8 were overexpressed upon pathogen infection [55,57] with GSTF8 also being overexpressed under H_2_O_2_ accumulation [80]. Furthermore, GSTF11 was observed to directly interact with SA, suggesting its activity being modulated by SA [81]. The underexpression of GSTF11 in the infested group suggested that the SA signalling mechanism might be suppressed by other hormones, possibly by ABA, due to the antagonistic nature of both signalling mechanisms [82]. PGM1 is involved in stomatal movement and vegetative growth. Deletion mutant assay analysis on *A. thaliana* found that a PGM1 deficient mutant had retarded growth and hyposensitive response to ABA-induced stomatal closure [83]. Apart from that, PGM1 gene was uniquely expressed under drought stress in vitro [84] and overexpressed under *Blumeria graminis* fungal infection [85]. These previous findings including this study suggest that the increased PGM1 protein expression is more likely due to OP acclimatising itself to RPW. This was corroborated by the observation that the overexpression only occurred in the sixth week post-infestation where the infestation stage was already significant.

### 3.4. Docking

The binding affinity for most putative interactions was lower than the binding affinity between the corresponding template and its ligand. This is perhaps due to the RPW ligands being smaller in size compared to the template ligands. The binding affinity was calculated based on the sum of hydrogen, Van der Waals and ionic interactions [86]. A smaller molecule forms fewer interactions with the protein, resulting in a lower binding affinity.

The five proteins with predicted putative interaction with RPW ligands of interest were LRR-FEI1, At5g58300, NIK, PSKR2, and At2g26730. Currently, the function of At5g58300 and At2g26730 are unknown. Thus, it is difficult to infer that these two proteins may mediate the OP-RPW interaction. LRR-FEI1 is involved in mediating cell wall homeostasis that results in root and hypocotyl growth [87]. As the identified role of LRR-FEI1 is not related to the plant’s defence, this receptor too is unlikely to mediate the OP-RPW interaction. NIK was reported in a study to recognise NSP produced from geminivirus and subsequently, induce tomato plant’s defence [88]. However, NIK also mediated defence against a different virus, cabbage leaf curl virus infection in *A. thaliana* [89]. These findings suggest that the same receptor can recognise multiple ligands or evolve differently according to species, resulting in the ability to recognise a different type of ligand. In either case, it is possible in OP that NIK plays a vital role in recognising the RPW ligand and subsequently mediating defence. For PSKR2, this receptor is vital in modulating seed development, fertilisation, osmotic stress, and bacterial infection response [90,91,92]. In addition, this receptor mediates ABA biosynthesis [91] which is not detected in all other modelled receptors except for PSKR1. Although PSKR2 has been established to modulate growth, it is possible that PSKR2 in OP plays an important role in recognising insect’s ligands such as those from RPW and activating the SAR mechanism via activation of ABA biosynthesis and signalling. It must also be noted that the other three receptors (i.e., LRR-FEI1, At5g58300, and At2g26730) may mediate RPW-OP interaction, especially LRR-FEI1 and At5g58300, because the docking site overlaps with the template ligand. However, the current literature lacks evidence to support them.

### 3.5. Abscisic Acid Driving Systemic Response

Overall, the trends of OP protein expressions under the three categories of interests largely resembled water-deficiency status. Moreover, a significant proportion of proteins involved in the processes of interest were associated with ABA response, with a few being associated with SA and MeJA. This study did not quantify plant hormones under RPW infestation. Nevertheless, a previous research reported that date palm (*Phoenix dactylifera*) showed elevated ABA, SA, and JA hormone levels [93], which corroborated the findings of this study. The digging activity by RPW destroys the vascular system of the host tree, impairing water and nutrient transport [6]. Consequently, the infested host trees exhibit protein expression patterns similar to drought-like stress.

Previous proteomics and transcriptomics experiments on the impact of RPW herbivory reported similar expression patterns to this study. Proteomics analysis of infestation on *P. dactylifera* reported HSP70 and HSP90 being overexpressed [94]. Similarly, under drought stress, HSP70 genes were upregulated in OP [95]. A transcriptomics study on RPW-infested *P. canariensis* found JA and ABA to be the primary drivers for infestation response based on the overexpression of JA biosynthesis-related genes (e.g., allene oxide synthase and 12-oxophytodienoate reductase 2 (OPR2)) and ABA response genes (e.g., HVA22K, C3HC4-type zinc-finger (RING finger), and two glycosyltransferases, rab-like GTPase activators, myotubularins (GRAM) domain-containing proteins) [96].

**Figure 6:**
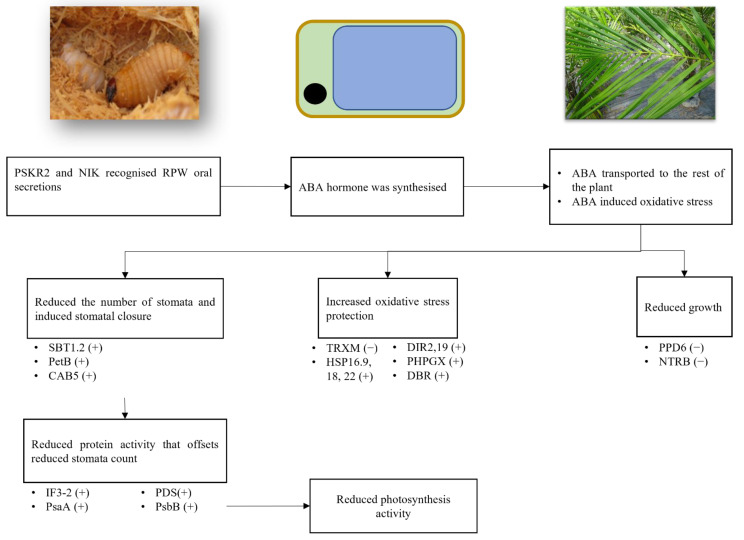
Summarised interaction between RPW and OP. (+): increased protein expression; (−): reduced protein expression. PSKR: phytosulfokine receptor; CAB5: chlorophyll ab binding protein; DBR: 2-alkenal reductase (NADP (+)-dependent); DIR2: Dirigent protein 2; DIR19: Dirigent protein 19; HSP16.9: 16.9 kDa class I heat shock protein 2; HSP18: 18.1 kDa class I heat shock protein; HSP22: 22.7 kDa class IV heat shock protein; IF3-2: translation initiation factor IF3-2, chloroplastic isoform X1; NTRB: NADPH-dependent thioredoxin reductase; PDS: 15-cis-phytoene desaturase; PetB: cytochrome b6; PHGPX: probable phospholipid hydroperoxide glutathione peroxidase; PPD6: Psbp domain-containing protein 6; PsaA: photosystem I P700 apoprotein; PsbB: photosystem II CP47 chlorophyll apoprotein; SBT1.2: subtilisin-like protease SBT1.2; TRXM: thioredoxin M-type.

Overall, the process of OP response to infestation is summarised in Figure 6. RPW larva secreted compounds may activate the ABA biosynthesis and the subsequent signalling via interaction with the LRR receptor. Based on the docking analysis, it is likely that some of those receptors mediate ABA signalling. PSKR2 is the most likely to mediate this interaction as a previous study found these receptors to mediate ABA biosynthesis [91]. NIK may also mediate OP–RPW interactions. However, further research is needed to verify both receptors’ interactions with RPW ligands.

Following the activation of ABA biosynthesis, ABA will be transported to the rest of the OP including the leaves. The accumulation of ABA in leaves activates the expression of proteins related to photosynthesis, growth, and stress response. These lead to the suppression of photosynthetic activity and growth. Therefore, proteins that reduce stomata development (e.g., SBT1.2) and induce stomatal closure (e.g., PetB and CAB5) are overexpressed. Apart from that, ROS-scavenging proteins (e.g., PHGPX, HSP, and PDS) that maintain the plant organelle integrity and protein functions are also overexpressed to offset the ROS accumulation that often follows ABA accumulation. These biological shifts may cause a drop in photosynthesis, as observed in our previous research [6].

In addition, several proteins can be viable candidate biomarkers for detecting RPW-infested OP trees (Table 5). They were chosen based on being induced by ABA signalling and differentially expressed in the infested OP in the first and third week post-infestation when the physical symptoms were still absent [6]. Six proteins were filtered and only one protein was differentially expressed on the third week post-infestation. These proteins were all underexpressed under infested group. Thus, developing a molecular biomarker based on these proteins requires a threshold value of which it will give a positive signal of infestation when the protein biomarker level dropped below that value. However, establishing the threshold value requires absolute quantification of the biomarker under control, physical wounding, and RPW infestation conditions. This can be achieved by performing selected reaction monitoring of the leaf samples collected in this study [97]. The selection reaction monitoring could also serve as a validation study of the differentially expressed proteins reported in this research.

## 4. Materials and Methods

### 4.1. Artificial Infestation

**Figure 7:**
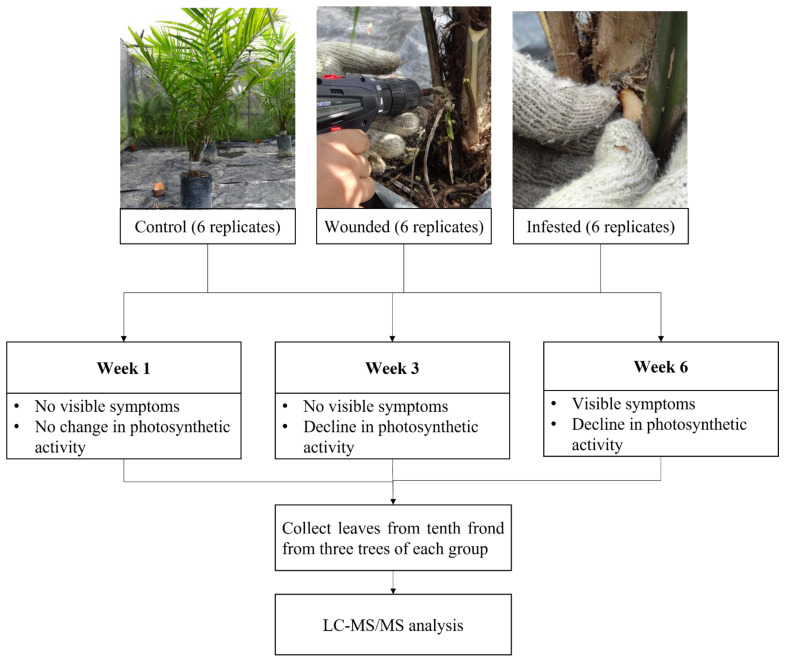
Summary of the RPW infestation experiment.

The RPW infestation was performed as described in our previous research investigating the impact of RPW on OP in terms of physical and physiological changes [6]. The experiment was performed in an enclosure covered by steel mesh to expose all OP trees to the ambient climate while preventing RPW from escaping. The trees were watered every two days. Hence, 18 two-year-old tissue cultured OP trees were divided into control, wounded (drilled three holes of 1.5 cm diameter and 5 cm depth), and infested (drilled three holes at the crown, with each hole was introduced with 1 RPW larva) groups with six trees for each group. During sampling, three OP trees were picked at random, and the leaf samples were collected from the tenth frond as it was the middle frond of the OP trees used in this study. The leaves sampled were also picked at random. Sampling was performed on the first (no physical damage and physiological changes), third (no physical damage but observed decline in photosynthetic activity), and sixth weeks (appearance of physical damage and decline in photosynthetic activity) post-infestation experiment for proteomic analysis. This process is summarised in (Figure 7).

### 4.2. Protein Extraction, SDS-PAGE and Peptide Digestion

The protein extraction protocol was performed following an optimised protein extraction method for OP leaves [98]. First, 1 g of ground sample powder from each of the three sampled OP trees from the control, wounded, and infested groups was used for each extraction procedure. The polyacrylamide gel for SDS-PAGE was prepared according to an established method by Laemmli [99] and the electrophoresis step was performed using Mini-PROTEAN following the manufacturer’s manual (Bio-Rad, Hercules, USA). The polyacrylamide gel was prepared with 12.5% acrylamide for the separating layer and 4% for the stacking layer. An equivalent volume of 100 µg of each OP sample extracts was mixed with 5X SDS loading dye buffer (10% (w/v) SDS, 10 mM dithiothreitol, 20% (v/v) glycerol, 0.2 M Tris-HCl (pH 6.8) and 0.05% bromophenol blue) at a ratio of 1:5. The SDS-PAGE was run at 75 V for 5 min to stack the proteins in a single band. Each band was cut out of the gel and chopped into cubes with approximately 1 mm^3^ dimension. The cubes were discoloured and digested with Trypsin following the previous method by Shevchenko [100].

### 4.3. Liquid Chromatography-Tandem Mass Spectrometry (LC-MS/MS)

The MS spectra generation was carried out by the Malaysian Genome Institute (MGI). Each extracted peptide was analysed with three technical replicates using Dionex nano RSLC LC-MS coupled LTQ Orbitrap Fusion (Thermo Fisher, Bremen, Germany) and Thermo Xcalibur MS as the system controller. First, 1 µL of each peptide replicate was injected onto a reversed phase on an EASY Spray Column Acclaim PepMap C18 100 A°, 75 µm id × 15 cm with 2 µm particle size. The mobile phase consisted of 0.1% formic acid in water (A) and 0.1% formic acid in acetonitrile (B). The eluted peptides were separated using a linear gradient 5% B at *t* = 0, 7% B at *t* = 5 min, 25% B at *t* = 90 min, 60% B at *t* = 108 min, 95% B at *t* = 113–123 min, and 2% B at *t* = 125–135 min. The total sample running time was 135 min with a constant flow of 0.3 μL/min [101].

The MS/MS data acquisition was performed in the electrospray ionisation positive mode, with charge state + 2, capillary temperature 275 °C, and spray voltage at 1.6 kV. Full MS scan was performed in the range from 310 to 1800 m/z acquired at 120,000, at an automatic gain control (AGC) target of 4 × 10^5^ and at a maximum injection time of 50 ms. Precursor ions with monoisotopic m/z and charge between 2 and 7 were selected for analysis. Selected precursors were filtered in 20-s isolation window at the threshold value of 5000. MS/MS spectra were analysed using Ion Trap MS/MS using the following parameters: high energy collision-induced dissociation (HCD) with normalised collision energy (NCE) set to 20% with 1.6 m/z isolation windows at targeted AGC 1.0 e^2^ and maximum injection at 250 ms.

### 4.4. Peptide Identification and Quantification

The data acquired from XCalibur software were processed by MaxQuant version 1.6.3.4 (http://maxquant.org; Date accessed: 20 December 2020) and searched by the Andromeda search engine [101]. The proteome sequence data from the OP proteome (NCBI taxon id:51953) was used as the reference proteome. The MaxQuant parameters were kept at default according to the MaxQuant documentation [102].

### 4.5. Statistical Analysis

Perseus statistical software (version 1.5.2.6) was used to perform statistical analysis from the MaxQuant output file. The MaxQuant data were filtered for protein identifications based on methods outlined by the software documentation [103]. Proteins that were successfully present in all three replicates were chosen for statistical analysis. One-way ANOVA followed by Tukey’s multiple comparison test with *p* < 0.05 and Log_2_ ratio > 1.5 or < −1.5 among the wounded and infested groups (W/I), control and wounded groups (C/W), and control and infested groups (C/I) were calculated to narrow down the differentially expressed proteins

### 4.6. Protein Enrichment Analysis and Literature Searches

For KEGG pathway enrichment analysis, differentially expressed proteins were uploaded to KOBAS version 3.0 (http://kobas.cbi.pku.edu.cn/; Date accessed: 5 January 2021). Further literature and UniProt database searches were performed on each protein. These proteins were categorised into photosynthesis, growth, and stress response. Proteins that did not fit the three categories were excluded. We also investigated their relationship with plant hormones, such as jasmonic acid (JA), salicylic acid (SA), and abscisic acid (ABA).

### 4.7. Receptor Protein and Ligand Selection

OP proteome sequences from the NCBI database were screened for potential receptors. Each protein sequence was analysed with TMHMM (version 2.0; Date accessed 5 March 2021), SignalP (version 5.0; Date accessed: 5 March 2021), WoLF PSORT (https://wolfpsort.hgc.jp/; Date accessed: 5 March 2021) and HMMER (version 3.3.2; Date accessed: 1 April 2021). TMHMM was used to predict the presence along with the number of transmembrane helices [104]. SignalP predicted the presence of signal peptides. WoLF PSORT predicted the localisation of the protein in the membrane [105]. HMMER searched for the leucine-rich repeat (LRR) domain, which was the only domain found to mediate insect herbivory-induced defence response in plants [21].

The selected proteins were modelled using the FunMod modelling platform [106]. HHsearch was used to search for template structures [107]. MODELLER (version 10.1) was used to model the protein sequences [106,108]. Only protein models with GA341 score > 0.7 [109] and normalised discrete optimised protein energy (nDOPE) < 0 [110] were selected for docking analysis. The selected models were listed in Table S4.

Candidate ligands were acquired from a previous metabolomic research on RPW secretions [20]. Literature research was performed on each compound correlated with inducing the plant immune response. The three-dimensional molecular structure for the selected ligands was downloaded from the PubChem database (www.pubchem.ncbi.nlm.nih.gov; Date accessed: 14 March 2021).

### 4.8. Docking Analysis

**Figure 8:**
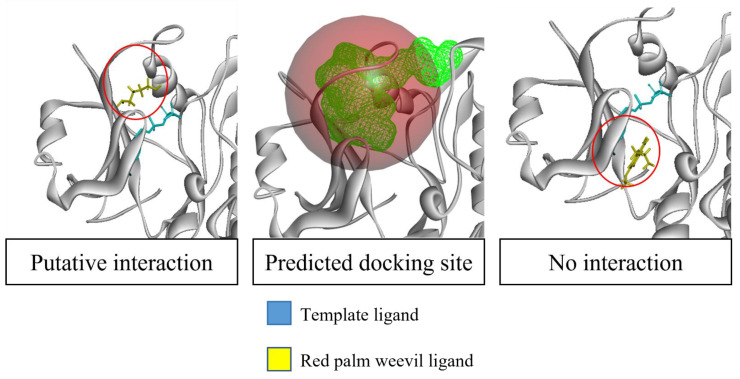
Method of determining putative interactions. Docking site was predicted using BIOVIA Discovery. Putative interaction is declared when the RPW ligand is positioned close to the template ligand’s binding position and positioned within the predicted docking site. If the RPW ligand is far from the template ligand or positioned outside the predicted docking site, then there is no interaction declared.

PyRx molecular docking software (www.pyrx.sourceforge.io; Date accessed: 1 April 2021) was used to perform high-throughput docking [111]. Protein model and ligand structures were loaded into the software, and the docking process was performed according to the manual [112]. The docking position with the highest binding affinity was chosen as the putative docking site for the ligand. Using BIOVIA Discovery Studio Visualiser software (Dassault Systemes), the protein template structure was superimposed to the respective protein models. The binding position of the template’s ligand was compared to RPW compounds’ positions. The binding positions of RPW ligands that shared a similar predicted docking position were declared as putative interactions. This process can be visualised in (Figure 8). A representative of each template that had the most putative interactions with RPW ligands was selected for further analysis.

## 5. Conclusions

Proteomic analysis of RPW-infested OP trees found many differentially expressed proteins to be responsive to ABA signalling. This suggests that the condition of RPW infestation is similar to the water-stress condition. The in silico molecular docking analysis predicted several candidate interactions between the OP receptors and RPW secretions. The most promising candidates were PSKR2, which was reported in the literature to mediate ABA biosynthesis, and NIK that mediated plant defence activation upon pathogen infection. Six proteins (PsaA, PsbB, REM, DIR19, PHGPX, CAB5) may be candidate biomarkers for developing a molecular-based system for detecting RPW-infested OP trees prior to symptoms manifestations

## Figures and Tables

**Table 1:** Top five most enriched KEGG pathway following KOBAS analysis with corrected *p* < 0.05.

Week	Comparison Group		
	Control/Infested	Wounded/Infested	Control/Wounded
**1**	Photosynthesis	Photosynthesis Ribosome Metabolic pathways Arachidonic acid metabolism	Glyoxylate and dicarboxylate metabolism Ribosome Nitrogen metabolism Propanoate metabolism
**3**	Glutathione metabolism	Biosynthesis of secondary metabolites Carbon metabolism Metabolic pathways Citrate cycle (TCA cycle) Photosynthesis-antenna proteins	Carbon metabolism Metabolic pathways Biosynthesis of secondary metabolites Citrate cycle (TCA cycle) Proteasome
**6**	Metabolic pathways Carbon metabolism Biosynthesis of secondary metabolites Citrate cycle (TCA cycle) Glutathione metabolism	Metabolic pathways Carbon metabolism Biosynthesis of amino acids Biosynthesis of secondary metabolites Protein processing in endoplasmic reticulum	Metabolic pathways Biosynthesis of secondary metabolites Tryptophan metabolism Glycolysis/Gluconeogenesis Limonene and pinene degradation

**Table 2:** Differentially expressed proteins in the infested group. These proteins were selected for having Log_2_ ratio between wounded (W) and infested group (I) of >1.5 or <−1.5, and having the Log_2_ ratio between the control and wounded group between −1.5 and 1.5. (+): Positively regulated by the hormone; (–): negatively regulated by the hormone.

NCBI Accession ID	Description	Abbreviation	Week	Function	Hormone Influence	Log2 FC W/I	Reference
XP_010905021.1	Translation initiation factor IF3-2, chloroplastic isoform X1	IF3-2	1	Photosynthesis	-	−1.622	[23]
YP_006073104.1	Photosystem I P700 apoprotein A1 (chloroplast)	PsaA	1	Photosynthesis	ABA(+)	−3.323	[24,25]
YP_006073130.1	Photosystem II CP47 chlorophyll apoprotein (chloroplast)	PsbB	1	Photosynthesis	MeJA(–); ABA(+)	−2.005	[25,26,27]
YP_006073134.1	Cytochrome b6 (chloroplast)	PetB	1	Photosynthesis	-	−2.962	[28]
XP_010912515.1	Dirigent protein 19	DIR19	1	Stress response	ABA(+,–); JA(+); MeJA(+)	−2.106	[29,30,31,32,33]
XP_010918555.1	Probable phospholipid hydroperoxide glutathione peroxidase	PHGPX	1	Stress response	JA(+); SA(+); ABA(+)	−2.365	[34]
XP_010905109.1	Remorin	REM	1	Stress response	ABA(+); SA(+)	−1.652	[35]
XP_010906967.1	Subtilisin-like protease SBT1.2	SBT1.2	1	Stress response	-	−2.776	[36,37,38,39]
XP_029118427.1	NADP(+) dependent 2-alkenal reductase	DBR	1	Stress response	-	−1.572	[39]
XP_010923778.1	Thiosulfate/3-mercaptopyruvate sulfurtransferase 2	3-MST	3	Growth	-	−1.515	[40]
XP_010930644.1	Thioredoxin M-type, chloroplastic	TRXM	3	Growth; photosynthesis	-	−2.021	[41,42,43]
XP_010908796.1	NADPH-dependent thioredoxin reductase	NTRB	3	Growth	-	1.787	[44,45]
XP_010936352.2	Chlorophyll a-b binding protein 5, chloroplastic	CAB5	3	Photosynthesis; Stress response	ABA(+)	−1.808	[46,47,48,49,50]
XP_010916973.1	15-cis-phytoene desaturase, chloroplastic/chromoplastic	PDS	3	Photosynthesis; Stress response	SA(+)	−1.635	[48,50]
XP_010925305.2	Extracellular ribonuclease LE	RNase LE	3	Stress response	-	2.389	[51,52]
XP_010906401.1	Psbp domain-containing protein 6, chloroplastic	PPD6	6	Growth	ABA(–)	1.512	[53,54]
YP_006073134.1	Cytochrome b6 (chloroplast)	PetB	6	Photosynthesis	ABA(+)	−1.546	[28]
XP_010912634.1	Dirigent protein 2	DIR2	6	Stress response	ABA(+,–); JA(+), MeJA(+)	−2.105	[29,30,32,33]
XP_010935284.1	Dirigent protein 7	DIR7	6	Stress response	ABA(+,–); JA(+), MeJA(+)	1.924	[29,30,32,33]
XP_010910894.1	Glutathione S-transferase F11	GSTF11	6	Stress response	SA(+)	2.431	[55,56,57]
XP_010912721.1	22.7 kDa class IV heat shock protein	HSP22	6	Stress response	ABA[+]; MeJA(+)	−1.844	[58,59,60]
XP_010925290.1	18.1 kDa class I heat shock protein	HSP18	6	Stress response	ABA[+]; MeJA(+)	−1.687	[58,59,60]
XP_010925996.1	16.9 kDa class I heat shock protein 2	HSP16.9	6	Stress response	ABA[+]; MeJA(+)	−1.660	[58,59,60]
XP_019708948.1	2,3-bisphosphoglycerate-independent phosphoglycerate mutase	PGM-I	6	Stress response	ABA(+)	2.581	[51]

**Table 3:** The red palm weevil (RPW) ligands with prevalence to inducing plant immune response based on literature searches.

Compound	Abbreviation	Reference
Putrescine	PUT	[61]
5-Methoxytryptamine	5-MT	[62]
***γ*** **-aminobutyric acid**	GABA	[63]
Aminooxyacetic acid	AAO	[64]

**Table 4:** Summarised docking binding affinity between OP receptor and RPW ligands. The binding affinity for ligands positioned away from template ligands were not shown (i.e., n/a). T: template, GABA: γ-aminobutrytic acid, 5-MT: methoxytriptamine, AAO: aminooxyacetic acid, PUT: putrescine.

Identity	Accession ID	Template Uniprot ID	Binding Affinity (kcal/mol)				
			T	GABA	5-MT	AAO	PUT
probable LRR receptor-like serine/threonine-protein kinase At5g45780 isoform X2	XP_010925612.1	3UIM	−6.9	n/a	−6.1	−3.8	n/a
protein NSP-INTERACTING KINASE 1	XP_010929457.1	3UIM	−6.9	n/a	n/a	n/a	n/a
LRR receptor kinase SERK2 isoform X1	XP_010937435.1	3UIM	−6.9	n/a	n/a	n/a	n/a
LRR receptor kinase SERK2 isoform X1	XP_010937436.1	3UIM	−6.9	n/a	n/a	n/a	n/a
LRR receptor kinase SERK2	XP_010939661.1	3UIM	−6.9	−4.8	−5.8	n/a	n/a
protein NSP-INTERACTING KINASE 1	XP_010942232.1	3UIM	−6.9	−4.4	n//a	n/a	−4.2
probable LRR receptor-like serine/threonine-protein kinase At5g45780 isoform X1	XP_029121312.1	3UIM	−6.9	n/a	−6.1	n/a	n/a
probable LRR receptor-like serine/threonine-protein kinase At3g47570	XP_010907375.1	4MN8	−5.2	n/a	n/a	−4.3	n/a
probable LRR receptor-like serine/threonine-protein kinase At3g47570	XP_010908730.1	4MN8	−5.2	n/a	−5.3	n/a	n/a
probable leucine-rich repeat receptor-like protein kinase At5g63930	XP_010933136.2	4MN8	−5.2	n/a	n/a	n/a	n/a
probably inactive leucine-rich repeat receptor-like protein kinase At3g28040 precursor	NP_001290509.1	4Z63	−6.8	n/a	n/a	−4.4	n/a
phytosulfokine receptor 2	XP_010929346.1	4Z63	−6.8	−4.1	−5.8	−3.7	−3.6
receptor-like protein kinase	XP_010930679.2	4Z63	−6.8	n/a	n/a	n/a	−3.9
LRR receptor-like serine/threonine-protein kinase GHR1	XP_010906523.1	5UV4	−8.2	n/a	n/a	n/a	n/a
probable leucine-rich repeat receptor-like protein kinase At5g63930	XP_010910517.1	5UV4	−8.2	n/a	−6.1	n/a	n/a
probable inactive receptor kinase At4g23740	XP_010915720.1	5UV4	−8.2	−3.8	−5.6	n/a	n/a
probable inactive receptor kinase At4g23740	XP_010915721.1	5UV4	−8.2	−3.8	−5.5	n/a	n/a
probable inactive receptor kinase At4g23740	XP_010925786.1	5UV4	−8.2	n/a	−5.6	n/a	−3.6
probable leucine-rich repeat receptor-like protein kinase At1g68400	XP_010933300.1	5UV4	−8.2	−4.2	−5.9	n/a	n/a
probable LRR receptor-like serine/threonine-protein kinase At1g53440	XP_010934669.1	5UV4	−8.2	n/a	n/a	n/a	n/a
putative kinase-like protein TMKL1	XP_010940648.1	5UV4	−8.2	n/a	n/a	n/a	−3.7
LRR receptor-like serine/threonine-protein kinase FEI 1 isoform X1	XP_010942956.1	5UV4	−8.2	n/a	−6	−4	n/a
probable inactive receptor kinase At4g23740	XP_019707070.1	5UV4	−8.2	n/a	−5.9	n/a	−4.1
probable inactive receptor kinase At4g23740	XP_029121337.1	5UV4	−8.2	n/a	n/a	n/a	−3.7
probable inactive receptor kinase At5g58300 isoform X2	XP_010931391.1	6BRJ	−8.9	n/a	−5.9	n/a	n/a
probable inactive receptor kinase At5g58300 isoform X2	XP_010931392.1	6BRJ	−8.9	n/a	−6	n/a	n/a
probable inactive receptor kinase At5g58300 isoform X2	XP_010931393.1	6BRJ	−8.9	n/a	−6	n/a	n/a
LOW QUALITY PROTEIN: receptor protein kinase TMK1	XP_010910643.2	6BSD	−9.5	n/a	n/a	n/a	n/a
probable inactive receptor kinase At1g48480	XP_010910915.1	6BSD	−9.5	n/a	n/a	n/a	n/a
probable inactive receptor kinase At2g26730	XP_010916177.1	6BSD	−9.5	n/a	−5.6	n/a	n/a
receptor-like protein 51	XP_010924732.1	6TME	−5.1	−4.1	n/a	−4.1	−3.5
probable LRR receptor-like serine/threonine-protein kinase At2g16250	XP_010936262.1	6TME	−5.1	n/a	n/a	n/a	n/a

**Table 5:** Proteins that could be harnessed as candidate biomarkers for molecular based RPW-infested trees detection. I: infested group; W: wounded group.

Accession ID	Identity	Abbreviation	Week	Log_2_ Ratio W/I
YP_006073104.1	Photosystem I P700 apoprotein A1 (chloroplast)	PsaA	1	3.323
YP_006073130.1	Photosystem II CP47 chlorophyll apoprotein (chloroplast)	PsbB	1	2.005
XP_010905109.1	Remorin	REM	1	1.652
XP_010912515.1	Dirigent protein 19	DIR19	1	2.106
XP_010918555.1	Probable phospholipid hydroperoxide glutathione peroxidase	PHGPX	1	2.365
XP_010936352.2	Chlorophyll ab binding protein 5	CAB5	3	1.808

## Data Availability

The mass spectrometry proteomics data have been deposited to the ProteomeXchange Consortium via the PRIDE [113] partner repository with the dataset identifier PXD028986.

## Supplementary Materials

The following are available online at https://www.mdpi.com/article/10.3390/plants10122574/s1, Figure S1: Predicted ligand docking positions on LRR receptor-like serine/threonine-protein kinase FEI 1 (LRR-FEI1) protein, Figure S2: Molecular interaction among 5-MT and AAO ligand on protein LRR-FEI1. (A) 3D interaction diagram between 5-MT (yellow) and LRR-FEI1 residue, Figure S3: Putative interaction between 5-MT ligand and protein ‘probable inactive receptor kinase At5g58300′ (At5g58300), Figure S4: Putative interaction between 5-MT ligand and protein ‘probable inactive receptor kinase At2g26730′ (At2g26730), Table S1: Differentially expressed proteins on the first week post-infestation, Table S2: Differentially expressed proteins on the third week post-infestation, Table S3: Differentially expressed proteins on the sixth week post-infestation.
