# Supplementary material for: Proteomics and Interspecies Interaction Analysis Revealed Abscisic Acid Signalling to Be the Primary Driver for Oil Palm’s Response against Red Palm Weevil Infestation
Source: Plants (Basel). 2021 Nov 25;10(12):2574. doi: 10.3390/plants10122574 (PMC8709180; doi:10.3390/plants10122574)
Supplement: Supplementary file 1 [file plants-10-02574-s001.zip › Supplementary Figures.pdf]

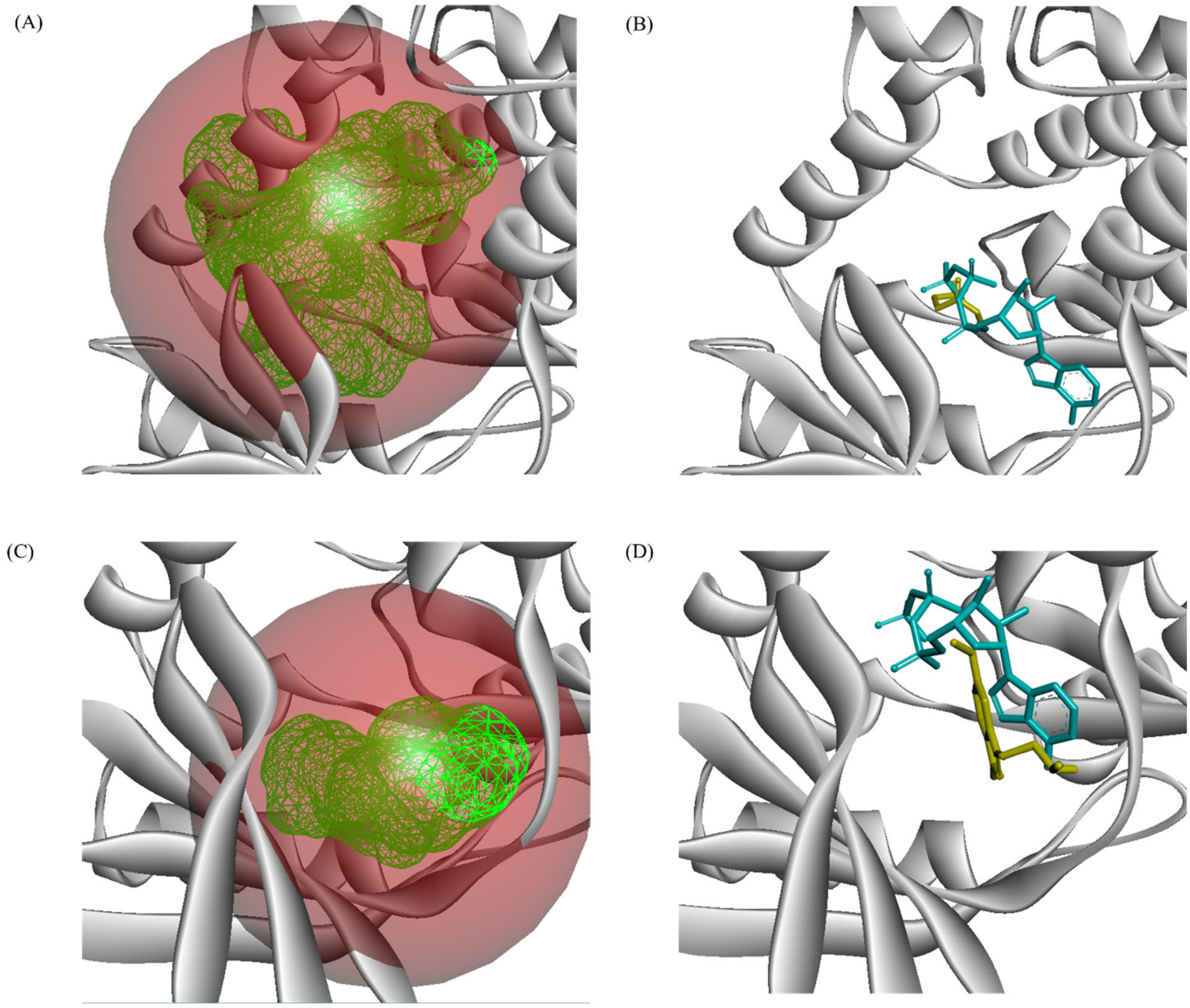

Figure S1. Predicted ligand docking positions on LRR receptor-like serine/threonine-protein kinase FEI 1 (LRR-FEI1) protein. (A) Predicted docking site highlighted in green mesh surface. (B) 5-MT ligand binding position (yellow) in comparison to template ligand (blue). (C) AAO ligand binding position (yellow) in comparison to template ligand. 5-MT: 5-methoxytriptamine; AAO: aminooxyacetic acid

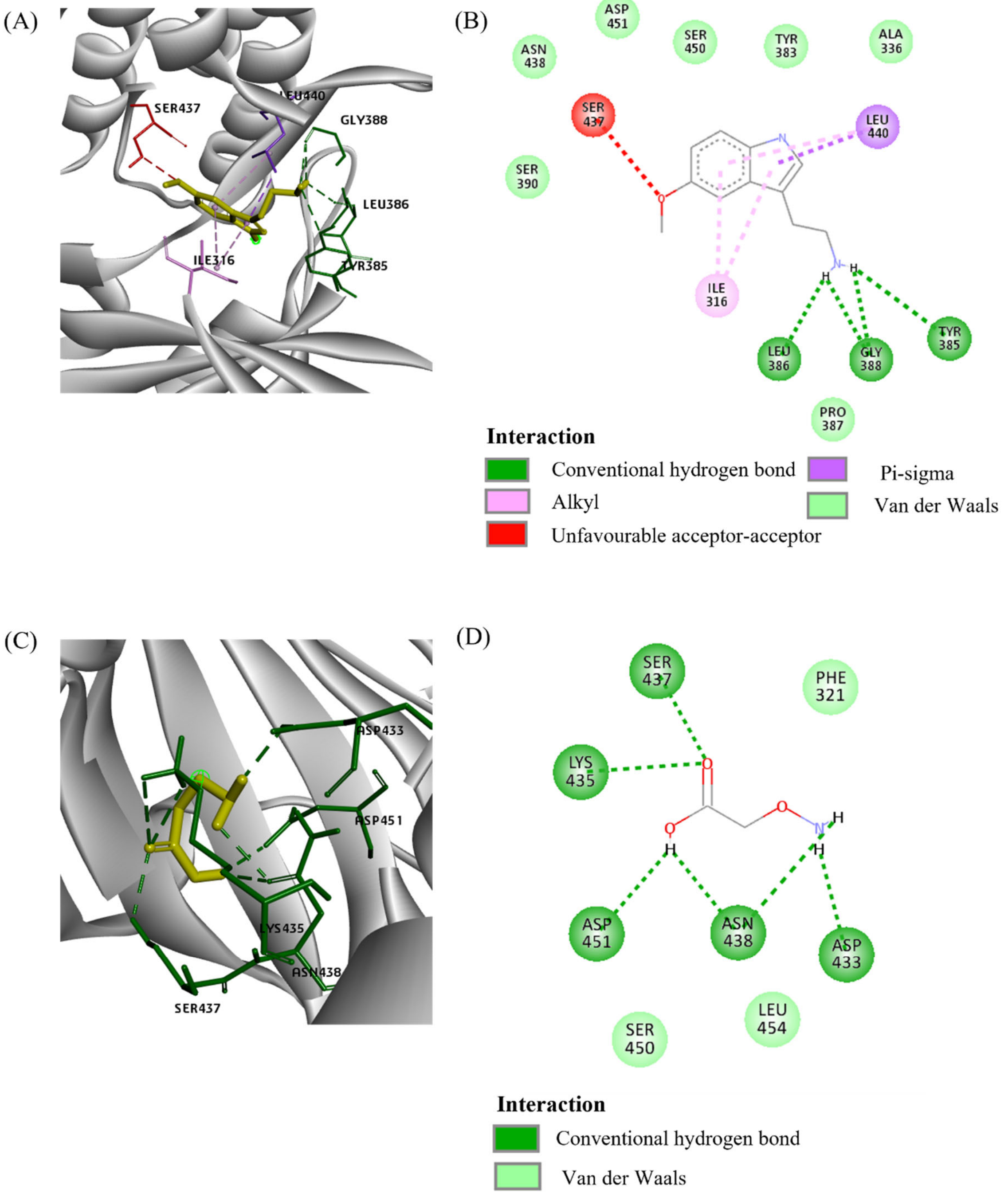

Figure S2. Molecular interaction among 5-MT and AAO ligand on protein LRR-FEI1. (A) 3D interaction diagram between 5-MT (yellow) and LRR-FEI1 residue. (B) 2D diagram interaction between 5-MT and LRR-FEI1. (C) 3D interaction diagram between AAO (yellow) and LRR FEI1. (D) 2D diagram interaction between AAO and LRR-FEI1. 5-MT: 5-methoxytriptamine; AAO: aminooxyacetic acid

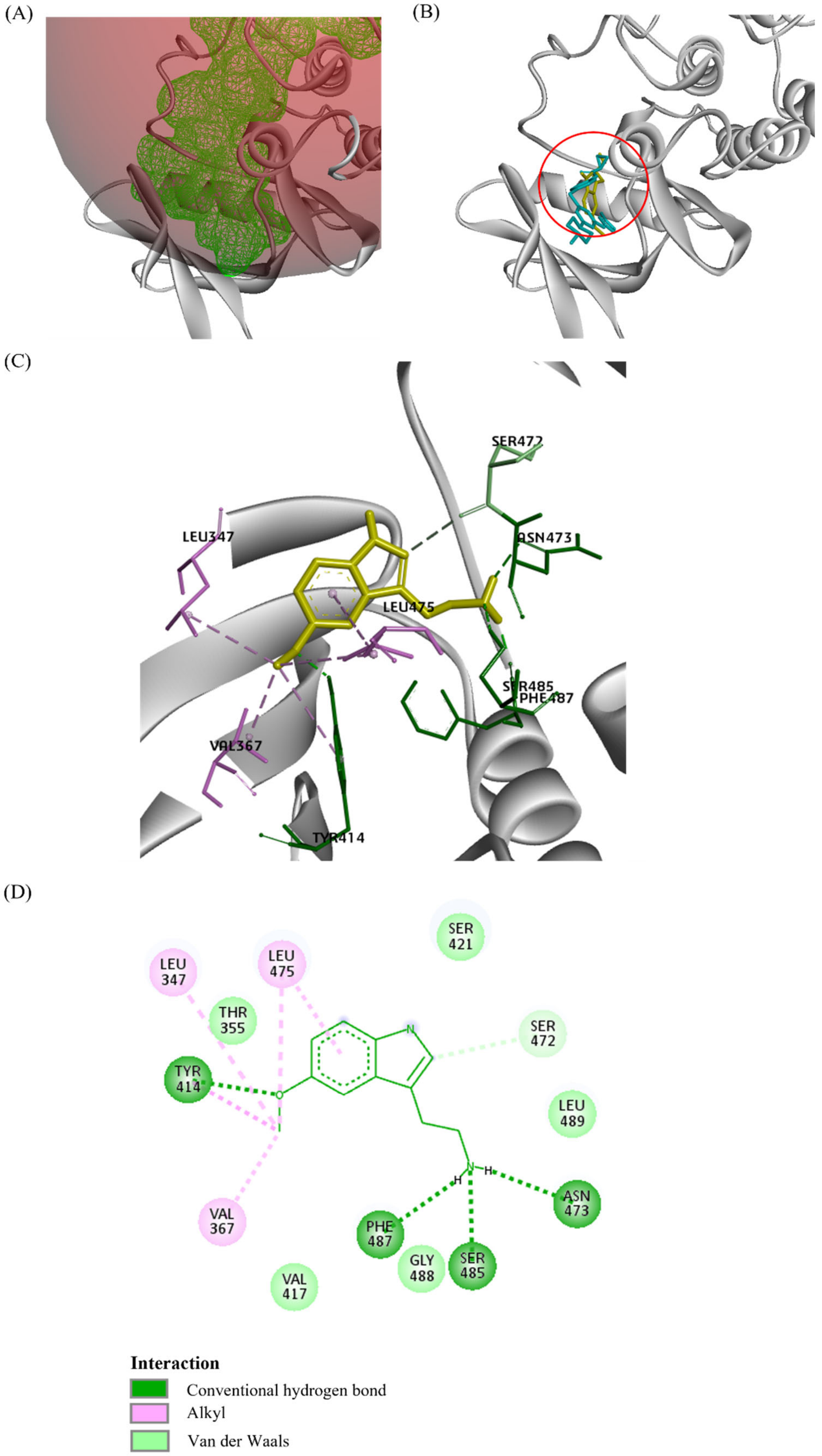
(A)
(B)
(C)
SER472
LEU347
ASN473
LEU475
SER485
PHE487
VAL367
TYR414
(D)
SER 421
LEU 347
LEU 475
THR 355
SER 472
TYR 414
LEU 489
ASN 473
VAL 367
PHE 487
GLY 488
SER 485
VAL 417
Interaction
Conventional hydrogen bond
Alkyl
Van der Waals

Figure S3. Putative interaction between 5-MT ligand and protein 'probable inactive receptor kinase At5g58300'(At5g58300). (A) Predicted ligand docking site highlighted in green mesh surface. (B) 5-MT ligand binding position (yellow) in comparison to template ligand (blue). (C) 3D interaction diagram between 5-MT (yellow) and At5g58300. (D) 2D interaction diagram between 5-MT and At5g58300. 5-MT: 5-methoxytriptamine.

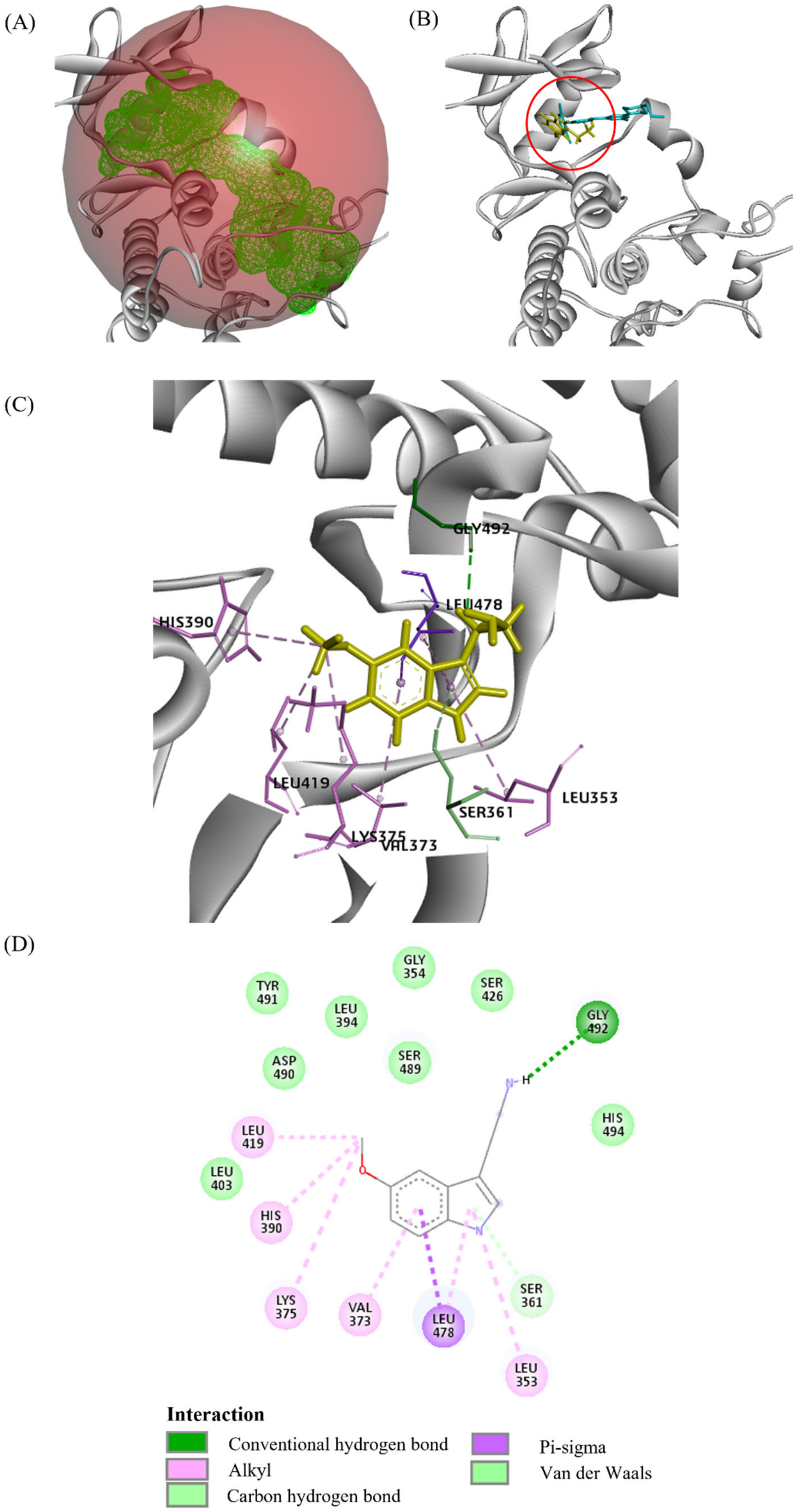

(A)
(B)
(C)
GLY492
LEU478
HIS390
LEU419
LYS375
VAL373
SER361
LEU353
(D)
GLY 354
TYR 491
SER 426
LEU 394
GLY 492
ASP 490
SER 489
HIS 494
LEU 419
LEU 403
HIS 390
LYS 375
VAL 373
LEU 478
SER 361
LEU 353
Interaction
Conventional hydrogen bond
Alkyl
Carbon hydrogen bond
Pi-sigma
Van der Waals

Figure S4. Putative interaction between 5-MT ligand and protein 'probable inactive receptor kinase At2g26730' (At2g26730). (A) Predicted ligand docking site highlighted in green mesh surface. (B) 5-MT ligand binding position (yellow) in comparison to template ligand (blue). (C) 3D interaction diagram between 5-MT (yellow) and At2g26730. (D) 2D interaction diagram between 5-MT and At2g26730. 5-MT: 5-methoxytriptamine
