# Supplementary material for: Proteomics and Interspecies Interaction Analysis Revealed Abscisic Acid Signalling to Be the Primary Driver for Oil Palm’s Response against Red Palm Weevil Infestation
Source: Plants (Basel). 2021 Nov 25;10(12):2574. doi: 10.3390/plants10122574 (PMC8709180; doi:10.3390/plants10122574)
Supplement: Supplementary file 1 [file plants-10-02574-s001.zip › Supplementary table caption.pdf]

Table S1. Differentially-expressed proteins among the control, wounded and infested oil palm (OP) groups with Log_2 ratio of > 1.5 or < −1.5 on the first week post red palm weevil (RPW) infestation.

Table S2. Differentially-expressed proteins among the control, wounded and infested oil palm (OP) groups with Log_2 ratio of > 1.5 or < −1.5 on the third week post red palm weevil (RPW) infestation.

Table S3. Differentially-expressed proteins among the control, wounded and infested oil palm (OP) groups with Log_2 ratio of > 1.5 or < −1.5 on the sixth week post red palm weevil (RPW) infestation.
